# Up-regulation of miR-210 by vascular endothelial growth factor in *ex vivo* expanded CD34+ cells enhances cell-mediated angiogenesis

**DOI:** 10.1111/j.1582-4934.2012.01557.x

**Published:** 2012-09-26

**Authors:** Mohamad Amer Alaiti, Masakazu Ishikawa, Haruchika Masuda, Daniel I Simon, Mukesh K Jain, Takayuki Asahara, Marco A Costa

**Affiliations:** a Department of Medicine Case Cardiovascular Research Institute Harrington-McLaughlin Heart & Vascular Institute, University Hospital Case Medical Center Case Western Reserve University School of MedicineCleveland, OH, USA; b Department of Regenerative Medicine Science, Tokai University School of MedicineIsehara, Kanagawa, Japan

**Keywords:** CD34+ cells, VEGF, miR-210, angiogenesis

## Abstract

*Ex vivo* culture has been proposed as a means to augment and repair autologous cells in patients with chronic diseases, but the mechanisms governing improvement in cell function are not well understood. Although microRNAs (miRs) are increasingly appreciated as key regulators of cellular function, a role for these factors in CD34+ cell-mediated angiogenesis has not been elucidated. Vascular endothelial growth factor (VEGF) was previously shown to induce expression of certain miRs associated with angiogenesis in endothelial cells and promote survival and number of vascular colony forming units of haematopoietic stem cells (HSCs). We sought to evaluate the role of VEGF in expansion and angiogenic function of CD34+ cells and to identify specific miRs associated with angiogenic properties of expanded cells. Umbilical cord blood CD34+ cells were effectively expanded (18- to 22-fold) in culture medium containing stem cell factor (SCF), Flt-3 ligand (Flt-3), thrombopoietin (TPO) and interleukin-6 (IL-6) with (postEX/+VEGF) and without VEGF (postEX/noVEGF). Tube formation in matrigel assay and tissue perfusion/capillary density in mice ischaemic hindlimb were significantly improved by postEX/+VEGF cells compared with fresh CD34+ and postEX/noVEGF cells. MiR-210 expression was significantly up-regulated in postEX/+VEGF cells. MiR-210 inhibitor abrogated and 210 mimic recapitulated the pro-angiogenic effects by treatment of postEX/+VEGF and postEX/noVEGF cells respectively. Collectively, these observations highlight a critical role for VEGF in enhancing the angiogenic property of expanded cells, and identify miR-210 as a potential therapeutic target to enhance CD34+ stem cell function for the treatment of ischaemic vascular disease.

## Introduction

The CD34+ cell population is an important target for therapeutic angiogenesis [1], but limited cell number and impaired function hinder its clinical application in patients with chronic diseases [2]. *Ex vivo* culture has been proposed as a means to augment and repair autologous cells, but the mechanisms governing improvement in cell function are not well understood. Although microRNAs (miRs), ∼21 nucleotide non-coding RNAs, are increasingly appreciated as key regulators of cellular function [3], a role for these factors in CD34+ cell-mediated angiogenesis remains largely unexplored. Certain miRs are promoted by VEGF in a Dicer-dependent fashion and have been implicated in endothelial cell-mediated post-natal angiogenesis [4–6]. The addition of VEGF to conventional haematopoietic stem cell expansion media [7] is appealing because of its positive role in cell survival and angiogenesis [8, 9], but the effects of such an approach to augment CD34+ cells are unknown. Therefore, we conducted a study to: (*i*) evaluate the impact of VEGF in *ex vivo* expansion of CD34+ cells, and (*ii*) identify specific miRs associated with angiogenic properties of *ex vivo* expanded CD34+ cells.

## Materials and methods

### Collection of CD34+ cells

Human cord blood (CB) was obtained from the University Hospitals Case Medical Center after IRB approval and written consent from donors. Total cord blood mononuclear cells were isolated by Histopaque 1077 (Sigma-Aldrich, Oakville, Ontario, Canada) density-gradient centrifugation. CD34+ cells were separated from mononuclear cells using CD34-bound microbeads and a magnetically activated cell sorter (autoMACS™; Miltenyi Biotec, Bergisch-Gladback, Germany) following the manufacturer's protocol. After separation, purity was determined by flow cytometry as described below.

### *Ex vivo* expansion of CD34+ cells

5 × 10^4^ CB-CD34+ cells in 2 ml of media were plated into each well of the six-well tissue culture dish (Primaria™; BD Falcon, Bedford, MA, USA) and cultured in a suspension manner using a serum-free expansion culture medium (CellGro® SCGM medium; CellGenix USA, Portsmouth, NH, USA) for 7 days. Expansion medium contained the four growth factors/cytokines: SCF (100 ng/ml), Flt-3 (100 ng/ml), TPO (20 ng/ml) and IL-6 (20 ng/ml), with or without VEGF (50 ng/ml). All growth factors and cytokines were purchased from Peprotech Inc. (Rocky Hill, NJ, USA).

### Characterization of fresh and post-expansion CD34+ cells

To confirm the purity and to characterize pre- and post- expansion cells, fluorescence-activated cell sorting (FACS) analysis was performed with BD™ LSR Cell Analyser (BD Biosciences, San Jose, CA, USA) and Cell Quest™ software (BD Biosciences) after staining with mouse anti-human monoclonal antibodies against surface markers: CD133-APC (clone 293C3; Miltenyi Biotec), CD34-PE (clone 581; Pharmingen, San Diego, CA, USA), CD45-FITC (Biolegend, San Diego, CA, USA), CXCR4-PE (Pharmingen), CD11b-PE (Biolegend), CD3-PE (Biolegend), CD19-PE (Biolegend). Dead cells were excluded from the plots on the basis of 7-AAD staining (Pharmingen). Cells were stained with monoclonal antibodies for 20 min. at 4°C following FcR blocking, washed twice using Hank's buffer containing 2% FBS, and analysed. Relevant isotype controls (IgG1-PE isotype control (Biolegend), IgG1-FITC (Biolegend), IgG2b-APC (Biolegend), and IgG1-APC (Biolegend)) were also included. In all samples, 10,000 events were acquired.

### MiRs expression analysis

Expression of miRs that have been previously associated with endothelial-mediated angiogenesis was determined in fresh and post-expansion cells (*n* = 3 in each group) using quantitative RT PCR. A quantity of 10 ng of total RNA was used for RT reactions from each sample following manufacturer's protocol (ABI kit). Reaction mixtures (15 μl) were incubated in a thermal cycler (Veriti® 96-Well Thermal Cycler; Applied Biosystems, Foster City, CA, USA) for 30 min. at 16°C, 30 min. at 42°C and 5 min. at 85°C and then maintained at 4°C. Quantitative PCR assays were performed using a TaqMan microRNA assay kit (Applied Biosystems). Real-time PCR was performed with a StepOnePlus™ Real-Time PCR System (Applied Biosystems). All reactions were incubated at 95°C for 10 min., followed by 40 cycles of 95°C for 15 sec., and 60°C for 1 min.; all were performed in triplicate. The RNU48 was used as a control to normalize differences in total RNA levels in each sample. A threshold cycle (Ct) was observed in the exponential phase of amplification, and quantification of relative expression levels was performed using standard curves for target genes and the endogenous control. Geometric means were used to calculate the ΔΔCt values and were expressed as 2^−ΔΔCt^. The value of each control sample was set at 1 and was used to calculate the fold of difference in the target gene.

### Transfection of expanded cells with miR-210 inhibitor and mimic

To silence or up-regulate miR-210, cells were transfected with specific Anti-miR™ miRNA Inhibitor or Pre-miR™ miRNA mimic, hsa-miR-210 (Applied Biosystems). On day 5 of expansion, cells were seeded in antibiotic-free expansion media and transfected with miRs at a final concentration of 160 nM using Lipofectamine 2000 (Invitrogen, Carlsbad, CA, USA). After 48 hrs incubation, cells were collected and washed twice with sterile phosphate-buffered saline (PBS). As a negative control of transfection, we used non-targeting scrambled oligonucleotide following same transfection method. To evaluate miR transfection efficiency, cells were transfected with FITC-conjugated miR-210 inhibitor (mercury LNA™ micro RNA Power inhibitor, hsa-miR-210, EXIQON, Woburn, MA, USA) and analysed using flow cytometry to count transfected cells. After transfection, obtained cells proceeded for gene expression analysis, flow cytometric analysis, tube formation assay and animal experiments as described.

### *In vitro* HUVEC tube formation assay

Pre- and post- expansion cells were applied to the tube formation assay by co-culturing with human umbilical vein endothelial cells (HUVECs) on Matrigel™ (BD Biosciences) to investigate their functional angiogenic contribution. 1 × 10^3^ cells from each group (preEX, postEX/+VEGF, and postEX/noVEGF) were co-cultured with 1.5 × 10^4^ HUVECs in 50 μl of EBM-2 complete medium with 2% FBS. A quantity of 50 μl of the cell suspension incubated at 37°C for 5 min. was applied onto Matrigel™ (50 μl/well) in 96-well plate. To evaluate miR-210 effect on angiogenic function of expanded cells, miR-210 inhibitor or mimic transfected cells were incubated with HUVEC following the same cell density. As a control, only HUVEC was cultured. After incubation for 18 hrs, a photomicrograph per well was taken under light microscopy (Leica DM IL LED with EC3 camera system, Buffalo Grove, IL, USA), then the number of tube formation was counted using Photoshop software.

### Mouse model of unilateral hind limb ischaemia and cell transplantation

The protocol was approved by the Case Western Reserve University School of Medicine Institutional Animal Care and Use Committee. Unilateral hind limb ischaemia was surgically induced as previously described [10]. Under anaesthesia with intraperitoneal xylazine (40 mg/kg) and ketamine (100 mg/kg), male 8- to 10-week-old NOD/SCID mice underwent left femoral artery ligation and transection at two points: proximally at inguinal ligament level and distally before bifurcation of popliteal and saphenous arteries. Two to six hours after surgery, 2.5 × 10^4^ cells of preEX, postEX/noVEGF, postEX/+VEGF, and cells transfected with miR-210 inhibitor, mimic or scrambled miRs suspended in 30 μl of PBS, were injected into adductor muscles. As a vehicle control, only PBS (30 μl) was injected into adductor muscles in the same manner (*n* = 5–6 per group).

### Perfusion imaging

Hind limb perfusion was measured with a laser Doppler perfusion imager system (Moor Instruments Ltd., Axminster, England) immediately and on day 14 after surgery. To account for variables, such as ambient light and temperature, the results were expressed as the mean flux ratio of perfusion in the left (ischaemic) *versus* the right (non-ischaemic) hind limb.

### Tissue preparation for histological immunofluorescent analysis

After completing blood flow measurements at 14 days, left calf muscles (ischaemic side) were harvested and immediately embedded in freezing compound (Triangle Biomedical Sciences, Inc., Durham, NC, USA). Transverse sections of 5-μm thick were made using the middle portion of calf muscle for subsequent staining procedures. For capillary density evaluation, samples were stained with anti-CD31 antibody, PECAM-1 (Santa Cruz Biotechnology, Santa Cruz, CA, USA) and labelled with fluorescent conjugated 2nd antibody (Alexafluora; Invitrogen). Capillaries, stained by CD31 with green fluorescent, were counted under a 200× magnification to determine the capillary density (number of capillaries per muscle bundle). Serial sections were cut at three different levels, and representative fields were analysed by counting the number of capillaries in each field. All samples were observed under a fluorescent microscope and images were taken using a digital camera system (Leica).

### Statistical analysis

Values were presented as mean ± S.E.M. One-way anova with Newman–Keuls *post-hoc* test were used to compare the experimental groups. Unpaired *t*-test was used for comparing two groups. A *P* < 0.05 was considered statistically significant.

## Results

### *Ex vivo* expansion and characterization of human CD34+ cells

**Fig 1:**
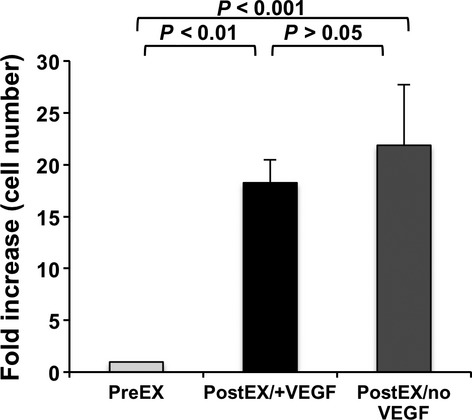
Fold increase in cell number after expansion with and without VEGF. Data presented as fold increase in cell number compared to preEX cells (adjusted to 1); *P* = 0.001.

**Fig 2:**
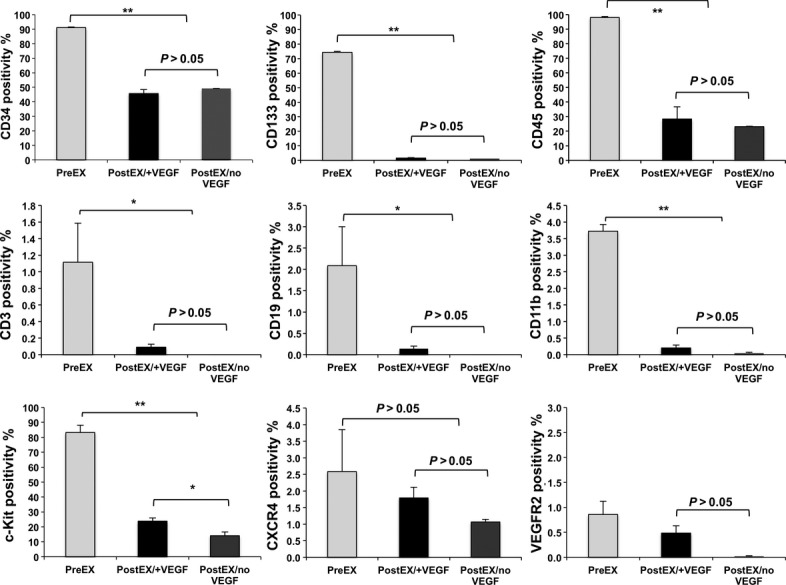
Representative flow cytometry data of preEX (fresh CD34+), postEX/+ VEGF, and postEX/noVEGF cells. Comparison of flow cytometry data showed decrease in CD34, CD133, and c-Kit in post-expansion cells, but no indication of differentiation into monocytic or lymphocytic lineages (CD11b, CD3 or CD19). There was low expression of CXCR4 and VEGFR2 in preEX cells and further decrease in both expansion groups. *P* <0.01 for CD34, CD133, c-Kit, CD11b, CD14 and CD45, *P* < 0.05 for CD3, CD19, and VEGFR2, and *P* > 0.05 for CXCR4. * and ** represents *post-hoc P* values of <0.05 and <0.01, respectively.

We first determined the expansion yield of CD34+ cells in medium containing four cytokines TPO, Flt3 ligand, SCF, and IL6 with (postEX/+VEGF) and without VEGF (postEX/noVEGF). CD34+ cell expansion was similar in both groups (Fold increase: 18.26 ± 2.24 *versus* 21.87 ± 5.85, respectively, *P* = 0.655) (Fig. 1). To characterize cells after expansion, we performed FACS analysis for CD34 positivity and other HSC/progenitor, monocytic and lymphocytic lineage markers (Fig. 2). CD34 positivity was 46% and 49% (*P* = n.s.) for postEX/+VEGF and postEX/no VEGF groups respectively (91% for freshly isolated cells) (Fig. 2). *Ex vivo* culture also resulted in significantly decreased CD133 and c-Kit positivity. There was modestly higher expression of c-Kit in the postEX/+VEGF group. FACS analysis suggested no differentiation into monocytic or lymphocytic lineages as indicated by the extremely low or absent expression of CD11b, CD3 and CD19.

### *Ex vivo* expansion in VEGF-enriched medium enhances neovascularization

**Fig 3:**
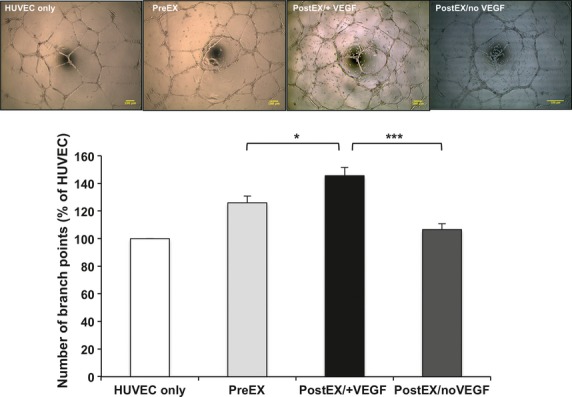
Enhanced HUVEC *in vitro* tube formation by CD34+ cells expanded in VEGF-enriched medium. Pre-or post- expanded CD34+ cells were washed and incubated with HUVEC in 96-well plate coated with matrigel. Upper panel shows representative images of different groups (×40; scale bar = 100 μm). Lower panel represents percentage increase in number of branch points compared to HUVEC alone. Data show significant increase in tube formation in postEX/+VEGF group (*P* < 0.0001). *, *** represents *post-hoc P* values of <0.05, and <0.001 respectively.

**Fig 4:**
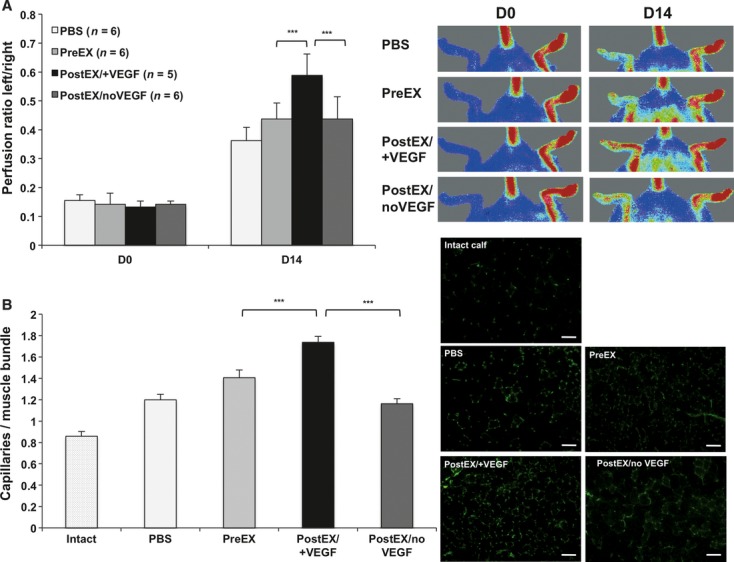
CD34+ cells expanded in VEGF-enriched medium promote tissue perfusion and capillary density in mice ischaemic hindlimb. PBS or 2.5 × 10^4^ cells were injected into ischaemic limb. Graphs show significant increase in tissue perfusion (A; *P* < 0.0001) and capillary density (B; *P* < 0.0001) in postEX/+VEGF group. Representative images of tissue perfusion and capillary density in calf muscle assessed by CD31staining (×200; scale bar = 200 μm) are shown in the right upper and lower panels, respectively. *** represents *post-hoc P* value of <0.001.

We first preformed the *in vitro* HUVEC tube formation assay to evaluate the effect of VEGF on the pro-angiogenic response by expanded cells. PostEX/+VEGF cells significantly improved *in vitro* tube formation in matrigel compared to both preEX and postEX/noVEGF groups (Fig. 3). We next compared the three cell groups in the mouse hind limb ischaemia model to further determine their *in vivo* neovascularization. Transplantation of postEX/+VEGF cells in the ischaemic hindlimb significantly improved tissue perfusion and increased capillary density compared to preEX and postEX/noVEGF cells (Fig. 4).

### VEGF augments miR-210 expression in expanded CD34+ cells

**Fig 5:**
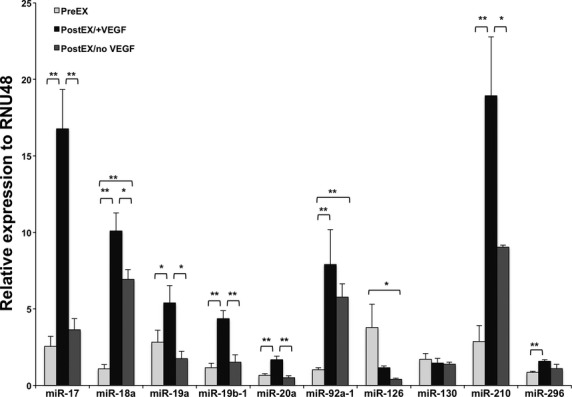
Expression profile of various miRs associated with angiogenesis. Representative graph of quantitative PCR data for selected miRs in PreEX (fresh CD34+), PostEX/+VEGF, and PostEX/noVEGF cell groups (*n* = 3 donors). *P* <0.01 for miR-17, 18a, 19b-1, 20a, 92a-1 and 210, *P* < 0.05 for miR-19a, 126 and 296 and *P* > 0.05 for miR-130. * and ** represents *post-hoc P* values of <0.05 and <0.01 respectively. RNU48 used as reference gene.

MiRs are increasingly appreciated as essential regulators of numerous cellular processes; however, their role in CD34+ cell-induced angiogenesis is not completely understood. VEGF was previously reported to induce expression of certain miRs associated with endothelial angiogenesis [6]. We therefore sought to identify specific miRs associated with angiogenic properties of *ex vivo* expanded CD34+ cells. Cells expanded in VEGF-containing medium altered the expression levels of various miRs (Fig. 5). VEGF treatment enhanced the expression of miRs, such as 17-92 cluster and -296 in expanded cells, similarly to previously reported effects in endothelial cells [6]. We found no change in miR-126 and -130a expression; miRs that were shown to be pro- angiogenic [11–13]. However, we found significant and robust increase in miR-210 level in postEX/+VEGF cells (Fig. 5). We focused our attention on miR-210 given its known anti-apoptotic and pro-angiogenic effects [14], but heretofore-unrecognized role in CD34+ cells.

### miR-210 silencing reduced postEX/+VEGF cell-mediated angiogenesis

**Fig 6:**
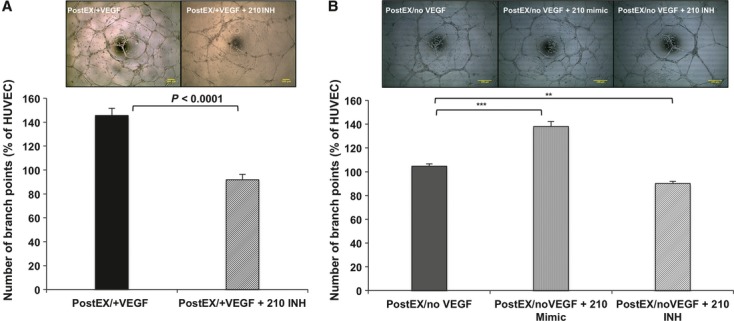
MiR-210 is essential for the pro-angiogenic effects of expanded CD34+ cells on HUVEC *in vitro* tube formation assay. Post-expansion cells were transfected with miR-210 inhibitor or mimic, washed and incubated with HUVEC in matrigel coated 96-well plate. MiR-210 inhibitor resulted in significant decrease in number of branch points by cells expanded with or without VEGF (A: *P* < 0.0001 and B: *P* < 0.0001), whereas 210-mimic enhanced tube formation by postEX/noVEGF cells. Upper panel shows representative images of different groups (×40; scale bar = 100 μm). Lower panel represents percentage increase in number of branch points compared to HUVEC alone. **, *** represents *post-hoc P* values of <0.01, and <0.001 respectively.

**Fig 7:**
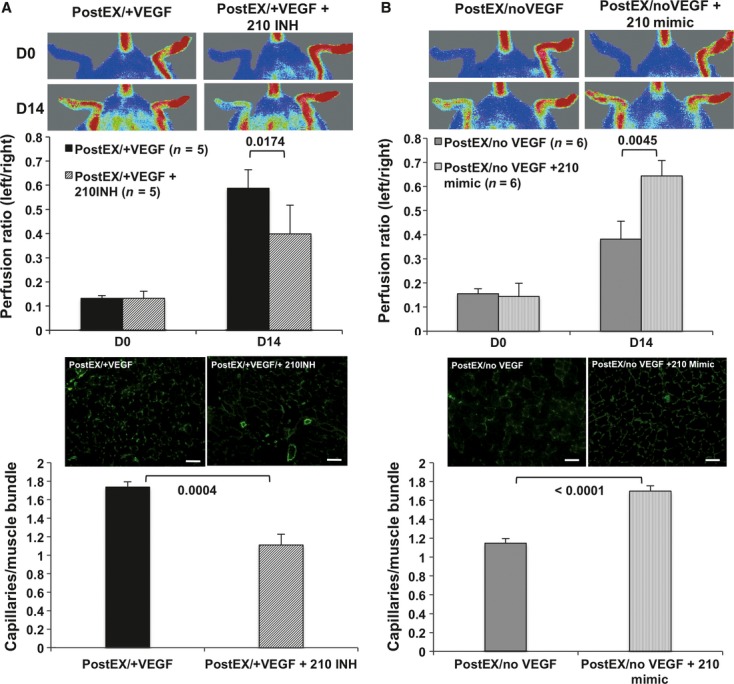
MiR-210 promotes tissue perfusion and capillary density by expanded CD34+ cells in mice ischaemic hindlimb. Expanded cells were transfected for 48 hrs with either miR-210 inhibitor or mimic on day 5. After washing, 2.5 × 10^4^ cells were injected into ischaemic limb. MiR-210 inhibition abrogated tissue re-perfusion (A, upper panel) and capillary density (A, lower panel) in postEX/+VEGF group, whereas mimic significantly improved tissue re-perfusion (B, upper panel) and capillary density (B, lower panel) in postEX/noVEGF. Representative images of tissue perfusion and capillary density in calf muscle assessed by CD31staining (×200; scale bar = 200 μm) are shown in the right upper and lower panels, respectively.

To test whether up-regulation of miR-210 is required for the enhanced angiogenic properties of postEX/+VEGF cells, we transfected cells with miR-210 silencing inhibitor on day 5 of *ex vivo* culture. Transfection efficiency was ∼60% (Fig. S1A), with specific ∼70% reduction in miR-210 level (Fig. S1B and C). Minimal effects of transfection with miRs on cell viability were confirmed by trypan blue dye exclusion test (∼87% *versus* ∼93% with no transfection). Additional control experiments were performed to exclude a deleterious effect of transfection procedure on cell function and expression of surface markers (Figs. S2 and S3). Inhibition of miR-210 in postEX/+VEGF cells was associated with reduced tube formation *in vitro* (Fig. 6A) and decreased tissue perfusion and capillary density *in vivo* (Fig. 7A).

### miR-210 mimic enhanced postEX/noVEGF cell-mediated angiogenesis

To further determine whether miR-210 is specifically involved in CD34+ cell-mediated angiogenesis independent of VEGF treatment, we transfected miR-210 mimic to CD34+ cells expanded in medium containing no VEGF. Similarly, 210-mimic was associated with enhanced *in vitro* tube formation (Fig. 6B) and increased tissue perfusion and capillary density *in vivo* (Fig. 7B).

## Discussion

Our study demonstrates an important role of VEGF in *ex vivo* augmentation of CD34+ cells that is miR-210-dependent. The addition of VEGF to a medium containing four cytokines commonly used for HSCs culture resulted in improved *in vitro* and *in vivo* angiogenesis, while preserving cell expansion yield. Importantly, cells expanded in VEGF-containing medium had a significant up-regulation in miR-210 expression (Fig. 5). Inhibition of miR-210 abrogated the pro-angiogenic effects of these cells (Figs 6A and 7A), and miR-210 mimic promoted cell-induced angiogenesis by cells expanded in VEGF-deficient medium (Figs 6B and 7B).

The importance of miRs in regulating the differentiation and fate of haematopoietic progenitor CD34+ cells has been previously described [15–18]. However, their role in angiogenic properties of *ex vivo* expanded CD34+ cells remains largely unexplored. VEGF is a central regulator of angiogenesis during development and ischaemia [19] and was shown to stimulate postnatal angiogenesis through enhancing expression of certain miRs in endothelial cells (EC) [6]. MiR17-92 cluster (17, 18a, 19a, 19b-1, 20a, 92a-1) was induced by VEGF, and it rescued EC proliferation and angiogenesis under VEGF stimulation after the loss of Dicer, an endoribonuclease required for generation and maturation of miRs [6]. In our study, we found significantly increased expression of this cluster after CD34+ cell expansion and VEGF treatment. Although this cluster has an important role in angiogenesis, cell survival and proliferation [20], its function is still incompletely understood. Individually, miR-17 has anti-proliferative properties [21, 22], whereas miR-92 is demonstrated to have anti-angiogenic effects [23], and miRs 18 and 19 are considered pro-angiogenic [24]. Additional studies are needed to explore the orchestrated mechanisms of this cluster in CD34+ cells and cell-induced angiogenesis.

We focused our attention on miR-210 given its established anti-apoptotic and pro-angiogenic effects [14], but heretofore-unrecognized role in CD34+ cells. MiR-210 regulates mitochondrial metabolism, cellular apoptosis and stem cell survival, as well as EC-mediated angiogenesis *in vitro* by regulating numerous targets, such as caspase-8-associated protein 2, protein-tyrosine phosphatase 1B, iron-sulphur cluster assembly proteins and ephrin-A3 mRNAs [14, 25]. MiR-210 also promotes migration of ECs in response to VEGF [5]. The expression of miR-210 in ECs was increased by hypoxia [5], but was not altered after VEGF exposure for 9 hrs [6]. Our study provides the first observation of increased levels of miR-210 induced by VEGF in CD34+ cells, and a new role for miR-210 in promoting CD34+ cell-mediated therapeutic angiogenesis *in vivo*.

Although CD34+ cells were cultured under normoxic conditions, it is possible that a state of relative hypoxia after 7-day culture led to the observed small increase in expression of miR-210 in postEX/noVEGF cells. These cells showed similar angiogenic capacity compared to pre-expanded CD34+ cells, suggesting the importance of VEGF exposure in augmenting pro-angiogenic properties of expanded CD34+ cells and raising the possibility that induction of miR-210 targets by VEGF may be required. It is possible that this increase in miR-210 expression may insufficiently compensate for the lack of other positive effects of VEGF stimulation, or for the phenotypic changes after *ex vivo* culture of CD34+ cells as demonstrated by FACS data (decreased expression of CD34, CD133 and c-Kit) (Fig. 2). Nonetheless, miR-210 silencing virtually eliminated whereas miR-210 mimic recapitulated the pro-angiogenic effects of VEGF stimulation on expanded cells (Figs 6 and 7). Collectively, these observations identify miR-210 as an important regulator of CD34+ cell-induced angiogenesis.

The number of cells transplanted into mice ischaemic hindlimbs in the present study was similar to weight-adjusted cell doses used in humans [26]. The 20–40 times lower number (2.5 × 10^4^ cells) compared to effective cell doses reported in previous pre-clinical studies [1, 27, 28] may explain the observed relative lower efficacy of pre-expanded (fresh) CD34+ cell transplantation. Importantly, postEX/+VEGF cell-induced *in vitro* and *in vivo* neovascularization was enhanced in spite of relatively low expression of CD34 and other known HSC and endothelial progenitor cell markers (Fig. 2).

Future investigation is warranted to dissect these pathways and explore whether the observed pro-angiogenic effects of miR-210 are cell-intrinsic [29] or operate through a paracrine exosome-mediated transfer of miR-210 from transplanted cells to the ischaemic tissue [30, 31]. Given the meaningful and sustained clinical benefit of CD34 cells in recent clinical trials [26], efforts to augment CD34+ cell function by targeting miR-210 are likely to be clinically impactful.

## Supporting information

**Figure S1** miR210 inhibitor transfection efficiency. Representative flow cytometric analysis of CD34+ cells transfected with unconjugated (control) or FITC-conjugated miR-210 inhibitor at 160 nM on day 5 of *ex vivo* expansion. Percentage of FITC-positive cells indicating ∼60% transfection efficiency after 48 hrs (**A**). Quantitative PCR data demonstrated specific (∼70%) inhibition of miR-210 (**B**), and no inhibition of other miRs (**C**). PostEX/+VEGF cells with (210INH) or without (control) specific miR-210 inhibitor were used for this experiment.

**Figure S2** Representative flow cytometry data showing no significant effect of miR-210 modulation on CD34, CD133, c-Kit or CXCR4 expression. PostEX/noVEGF cells were used as control (no transfection), or transfected with scramble miRs, miR-210 mimic, or miR-210 inhibitor. This data suggests that miR-210 is not involved in expression of these markers. Representative *P* values by anova are shown.

**Figure S3** Representative data for *in vitro* tube formation matrigel assay (**A**) and tissue perfusion 14 days post hind ischaemic hindlimb (**B**) showing no significant effect of transfection procedure on angiogenic properties of expanded cells. PostEX/noVEGF cells with and without (control) scramble miRs transfection were used for this experiment. (×40; scale bar = 100 μm for tube formation assay images).
