# Supplementary material for: Up-regulation of miR-210 by vascular endothelial growth factor in ex vivo expanded CD34+ cells enhances cell-mediated angiogenesis
Source: J Cell Mol Med. 2012 Sep 26;16(10):2413–21. doi: 10.1111/j.1582-4934.2012.01557.x (PMC3823435; doi:10.1111/j.1582-4934.2012.01557.x)
Supplement: Supplementary file 1 [file jcmm0016-2413-SD1.docx]

**Figure S1.** miR210 inhibitor transfection efficiency. Representative flow cytometric analysis of CD34+ cells transfected with unconjugated (control) or FITC-conjugated miR-210 inhibitor at 160 nM on day 5 of *ex vivo* expansion. Percentage of FITC-positive cells indicating ~ 60% transfection efficiency after 48 h (A). Quantitative PCR data demonstrated specific (~70%) inhibition of miR-210 (B), and no inhibition of other miRs (C). PostEX/+VEGF cells with (210INH) or without (control) specific miR-210 inhibitor were used for this experiment.
