# Supplementary material for: Up-regulation of miR-210 by vascular endothelial growth factor in ex vivo expanded CD34+ cells enhances cell-mediated angiogenesis
Source: J Cell Mol Med. 2012 Sep 26;16(10):2413–21. doi: 10.1111/j.1582-4934.2012.01557.x (PMC3823435; doi:10.1111/j.1582-4934.2012.01557.x)
Supplement: Supplementary file 2 [file jcmm0016-2413-SD2.docx]

**Figure S2.** Representative flow cytometry data showing no significant effect of miR-210 modulation on CD34, CD133, c-Kit or CXCR4 expression. PostEX/noVEGF cells were used as control (no transfection), or transfected with scramble miRs, miR-210 mimic, or miR-210 inhibitor. This data suggests that miR-210 is not involved in expression of these markers. Representative p values by ANOVA are shown.
