# Supplementary material for: Up-regulation of miR-210 by vascular endothelial growth factor in ex vivo expanded CD34+ cells enhances cell-mediated angiogenesis
Source: J Cell Mol Med. 2012 Sep 26;16(10):2413–21. doi: 10.1111/j.1582-4934.2012.01557.x (PMC3823435; doi:10.1111/j.1582-4934.2012.01557.x)
Supplement: Supplementary file 3 [file jcmm0016-2413-SD3.docx]

**Figure S3**. Representative data for *in vitro* tube formation matrigel assay (A) and tissue perfusion 14 days post hind ischemic hindlimb (B) showing no significant effect of transfection procedure on angiogenic properties of expanded cells. PostEX/noVEGF cells with and without (control) scramble miRs transfection were used for this experiment. (X40; scale bar = 100um for tube formation assay images).
